# Supplementary material for: Resolving the full spectrum of human genome variation using Linked-Reads
Source: Genome Res. 2019 Apr;29(4):635–45. doi: 10.1101/gr.234443.118 (PMC6442396; doi:10.1101/gr.234443.118)
Supplement: Supplemental Material [file supp_gr.234443.118_Supplemental_longranger-2.2.zip › longranger-2.2.tar.bz2!/lr2.2-public/lib/stan/cmdstan/stan/doc/api/html/namespace_eigen_1_1internal.html]

Stan: Eigen::internal Namespace Reference


|  |  |
| --- | --- |
|  | Stan  2.5.0  probability, sampling & optimization |


- Main Page
- Related Pages
- Namespaces
- Classes
- Files

- Namespace List
- Namespace Members

All Classes Namespaces Files Functions Variables Typedefs Enumerations Enumerator Friends Macros Pages

- Eigen
- internal

Classes

Eigen::internal Namespace Reference

(Expert) Product traits for algorithmic differentiation variables.
More...

|  |  |
| --- | --- |
| Classes | |
| struct | significant\_decimals\_default\_impl< stan::agrad::var, false > |
|  | Implemented this for printing to stream. More... |
|  | |
| struct | scalar\_product\_traits< stan::agrad::var, double > |
|  | Scalar product traits override for Eigen for automatic gradient variables. More... |
|  | |
| struct | scalar\_product\_traits< double, stan::agrad::var > |
|  | Scalar product traits override for Eigen for automatic gradient variables. More... |
|  | |
| struct | general\_matrix\_vector\_product< Index, stan::agrad::var, ColMajor, ConjugateLhs, stan::agrad::var, ConjugateRhs > |
|  | Override matrix-vector and matrix-matrix products to use more efficient implementation. More... |
|  | |
| struct | general\_matrix\_vector\_product< Index, stan::agrad::var, RowMajor, ConjugateLhs, stan::agrad::var, ConjugateRhs > |
|  | |
| struct | general\_matrix\_matrix\_product< Index, stan::agrad::var, LhsStorageOrder, ConjugateLhs, stan::agrad::var, RhsStorageOrder, ConjugateRhs, ColMajor > |
|  | |

## Detailed Description

(Expert) Product traits for algorithmic differentiation variables.


---

[ Stan Home Page ]

*© 2011–2014,
Stan Development Team.*
